# Reg3A (regenerating family member 3 alpha) acts as a tumor suppressor by targeting DMBT1 (deleted in malignant brain tumors 1) in gastric cancer

**DOI:** 10.1080/21655979.2021.1981800

**Published:** 2021-10-04

**Authors:** Liang Wang, Hongfang Tuo, Zhe Song, Wei Li, Yanhui Peng

**Affiliations:** a Department of Surgery, Hebei Medical University, Shijiazhuang, China; b Department of Surgery, Hebei General Hospital, Shijiazhuang, China; c The Second Department of General Surgery, Cangzhou Central Hospital, Cangzhou, China

**Keywords:** Regenerating family member 3 alpha, deleted in malignant brain tumor 1, gastric cancer, cell proliferation, tumor suppressor

## Abstract

Regenerating family member 3 alpha (Reg3A) encodes a pancreatic secretory protein that may be involved in cell proliferation or differentiation. However, the function and downstream regulatory mechanism of Reg3A in gastric cancer (GC) remains elusive. This study aimed to clarify the function and mechanism of Reg3A regulating cell proliferation in GC. The expression levels of Reg3A were confirmed in GC patients and cells using qRT-PCR and western blotting. TCGA datasets and clinical samples were used to explore the correlation between Reg3A and clinicopathologic features in GC. Cell viability, colony formation, and xenograft tumorigenesis assays were performed to detect the function of Reg3A on cell proliferation. Besides, we predicted the correlated genes of Reg3A by analyzing TCGA datasets, and further investigated the downstream regulatory mechanism of Reg3A in GC. Our results demonstrated that Reg3A is down-regulated in *vitro* and *vivo* (P < 0.05). Reg3A expression are negatively correlated with TNM classification (*P* < 0.001), lymph node (*P* < 0.001) in GC. Reg3A significantly suppresses cell proliferation in GC (P < 0.05). Bioinformatic analysis and experimental results confirmed that Reg3A positively regulates the expression of deleted in malignant brain tumor 1 (DMBT1, *P* < 0.05). Besides, Reg3A and DMBT1 all prolong the overall survival (OS, *P* < 0.01), post-progression survival (PPS, *P* < 0.05), and first progression survival (FP, *P* < 0.01). The function of Reg3A inhibiting cell proliferation is abolished by DMBT1 siRNA in GC (*P* < 0.05). In conclusion, Reg3A may act as a novel tumor suppressor by promoting DMBT1 expression, which may be a potential therapeutic target in patients with GC.

## 1. Introduction

The incidence of gastric cancer (GC) ranks fifth in the world, and the mortality rate of GC ranks third in developed countries [1,2]. GC was also a growing public health concern in low and medium human development index countries [3]. The risk factors of GC include helicobacter pylori infection, smoking history, chronic atrophic gastritis, and gastric surgery [4]. Currently, surgery is the most effective treatment for GC. However, nearly half of GCs relapse and develop into advanced disease after surgical resection, resulting in a relatively poor five-year survival rate for GC patients [5]. The past decade has seen the rapid development of molecular targeted therapy and immunotherapy in many cancers. The discovery of key targets will help improve clinical outcomes.

Regenerating family member 3 alpha (Reg3A) encodes a pancreatic secretory protein that may be involved in cell proliferation or differentiation [6]. Recently, Reg3A plays crucial roles in a variety of diseases, such as lupus nephritis [7], polymyositis and dermatomyositis [8], keratinocytes [9], and cancers [10]. In particular, there are a growing number of publications focusing on the function of Reg3A in human cancers, including GC [6], pancreatic cancer [11], colorectal cancer [12], hepatocellular carcinoma [13], and glioma [14]. Cho et al. revealed that tumor-stroma crosstalk promotes Reg3A expression that drives the progression of hepatocellular carcinoma [13]. In gastric cancer, previous studies have reported the effect of Reg3A on cell biological function. However, the results remain controversial, and the detailed mechanisms of Reg3A in GC remain unclear.

Deleted in malignant brain tumor 1 (DMBT1) is a member of the scavenger receptor cysteine-rich superfamily, which mediates cell-extracellular matrix interactions [15]. DMBT1 is highly unstable in cancer [16]. Extensive studies revealed that DMBT1 acts as a tumor suppressor in various types of cancer, such as lung cancer [15], breast cancer [17], digestive tract cancer [18], prostate cancer [19], gallbladder carcinoma [20], oral squamous cell carcinoma [21], and ovarian cancer [22]). In ovarian, Ma et al. revealed that DMBT1 suppresses cell proliferation, migration and invasion, and enhances the chemosensitivity of cisplatin [22]. In GC, a previous study reported that DMBT1 is downregulated in GC, and significantly suppresses cell proliferation in GC [23]. However, the molecular regulatory mechanism of DMBT1 is still unclear.

In the present study, we aimed to clarify the function and the regulatory mechanisms of Reg3A in GC. We explored the expression of Reg3A in GC and analyzed the correlation of Reg3A with clinical characteristics, such as TNM classification, lymph node, grade of differentiation, and survival time. Besides, We illuminated the functions and correlations of Reg3A and DMBT1 in GC. Considering the clues above, we hypothesized that Reg3A acts as a tumor suppressor by targeting DMBT1 in GC. So, we further explored the regulatory mechanism between Reg3A and DMBT1 in GC.

This study is the first to report the regulatory relationship between Reg3A and DMBT1. We hope that these data may provide novel biomarker candidates for the diagnosis and treatment of GC.

## 2. Materials and methods

### 2.1 Cell lines, cell culture, and clinical specimens

The human GC cells (SGC-7901, BGC-823, MGC-803, SNU638, AGS, NCI-N87) and human gastric epithelial cells (GES-1) were obtained from the Cell Bank of Shanghai Academy of Chinese Sciences. Forty paired clinical specimens (tumor and adjacent tissues) were provided by patients with GC in Hebei General Hospital (from June 2018 to June 2020). Inclusion benchmarks were: (1) newly histologically diagnosed stomach adenocarcinoma; (2) received no prior therapy before surgery; (3) underwent gastrectomy; (4) provided informed consent of using specimens. Exclusion benchmarks were: (1) tumor volume was insufficient for the analysis; (2) additional surgery after endoscopic resection; (3) no histories of other cancer. Ethical approval was obtained from Hebei General Hospital. AGS cell line was cultured in F12K medium. Other cell lines were cultured in RPMI 1640 medium. All mediums were supplemented with 10% fetal bovine serum and penicillin/streptomycin. Cells were cultured at 37°C under 95% air, 5% CO_2_.

### 2.2 Quantitative real-time PCR (qRT-PCR)

Total RNAs were extracted using Trizol reagent (Invitrogen, USA) by the manufacturer’s instructions. For complementary DNA synthesis, 1 μg total RNA was reverse transcribed using the Primescript RT reagent kit (Takara, Japan). Relative expression levels were detected using SYBR Green PCR Kit (Takara, Japan) and calculated using the 2^−ΔΔCT^ method. The parameters were used: 95°C for 60 sec, followed by 40 cycles of 95°C for 15 sec, 55°C for 15 sec, and 72°C for 20 sec. qRT-PCR primers were as follows: Reg3A forward: 5ʹ- AGCTACTCATACGTCTGGATTGG-3ʹ, Reg3A reverse: 5ʹ-CACCTCAGAAATGCTGTGCTT-3ʹ; DMBT1 forward: 5ʹ-CACCTCAGAAATGCTGTGCTT-3ʹ, DMBT1 reverse: 5ʹ-GCCACCAATCTGTCGTAGTAGAA-3ʹ.

### 2.3 Transfection

Reg3A siRNA, Reg3A overexpression vector, DMBT1 siRNA, and DMBT1 overexpression vector were synthesized by GenePharm (Shanghai, China), which sequences are as follows: Reg3A siRNA, 5ʹ- GCUGCUGUCUCAGGUUCAAGG-3ʹ(sense), 5ʹ-UUGAACCUGAGACAGCAGCAU-3ʹ (antisense); DMBT1 siRNA, 5ʹ-GGUGGAUGUGUAUUAUUAAGA-3ʹ (sense), 5ʹ-UUAAUAAUACACAUCCACCUU-3ʹ (antisense). The overexpression vector is GV492. Lipofectamine 2000 reagent (Invitrogen, USA) was used to transfect AGS or SGC-7901cells.

### 2.4 Cell viability

Cell viability was measured with Cell Counting Kit-8 (Dojindo, Japan). In brief, SGC-7901 and AGS cells were seeded in 96-well plates and incubated for 24 h, 48 h, or 72 h, respectively. Then, 10 µl CCK-8 solution was added to 100 µl medium and incubated for 1 h at 37°C. A microplate reader detected the optical density at 450 nm.

### 2.5 Cell proliferation

For colony formation assay, GC cells were seeded at 200 cells per well in 12-wells plates and were incubated at 37°C under 5% CO_2_ after transfection. About two weeks later, cells were fixed with 95% alcohol for 30 min at room temperature. Cells were stained with crystal violet (0.1%). Plaques were calculated with Image J software. Each assay was repeated three times.

### 2.6 Bioinformatics analysis

Cancer Hallmarks Analytics Tool (CHAT, https://chat.lionproject.net/) [24] was used to analyze the phenotype of DMBT1. GEPIA online tool (http://gepia.cancer-pku.cn/) [25] was performed to investigate the expression difference of Reg3A in GC. Kaplan Meier database (https://kmplot.com/analysis/) [26] was used to analyze the overall survival (OS), post-progression survival (PPS), first progression (FP), and relapse-free survival (RFS). LinkedOmics online tool (http://www.linkedomics.org/login.php) [27] was used to analyze the correlated genes of Reg3A in GC. Besides, the correlation of Reg3A or DMBT1 with the clinical characteristics was also investigated by LinkedOmics online tool.

### 2.7 Western blot analysis

Western blot assays were performed to detect the protein expression of Reg3A, Ki67, PCNA, and β-actin (ImmunoWay, USA). Cells were lysed with Lysis Buffer (CST, USA) on ice for 30 min, which was centrifuged at 14,000 rpm for 10 min at 4°C. The protein samples were normalized using a BCA assay kit (Shanghai, China). Equal amounts of protein lysates were electrophoresed on SDS-PAGE gels and then transferred onto nitrocellulose membranes. After blocked 1 h at room temperature, membranes were incubated with primary antibody overnight at 4°C. Membranes were then washed in PBST (PBS with 0.1% Tween) and incubated with horseradish peroxidase-labeled secondary antibody for 1 h at room temperature. After being washed in PBST three times, membranes were visualized by enhanced chemiluminescence reagents (KeyGen, China).

### 2.8 Xenograft tumorigenesis assays

Nude mice (BALB/c_nu/nu, 3–4 weeks old) were purchased from Beijing Vital River Laboratory Animal Technology Co. Ltd. The care and breeding of animals were performed in compliance with national guidelines. Mice were acclimatized to laboratory conditions (26°C, 12 h/12 h light/dark, 50% humidity, *ad libitum* access to food and water) for 1 week [28]. Mice were randomly divided into two groups (5 mice in each group). AGS cells or siReg3A AGS cells (5 × 10^6^/mouse) were resuspended in 100 µl PBS and then subcutaneously injected into each mouse. The tumor volume was measured with a caliper every three days. After thirty days, the mice were euthanized by cervical dislocation in deep anesthesia (isoflurane), and the tumors were surgically isolated and fixed for immunohistochemistry.

### 2.9 Immunohistochemistry

Tumor tissues were fixed using 4% paraformaldehyde for 24 h at 4°C. Paraffin sections were prepared according to standard procedure (including dehydrating using graded ethanol, vitrification by xylene, waxing, embedding, and section). Immunohistochemistry assays were performed using a one-step Immunohistochemistry kit (KeyGen, China). Paraffin sections were dewaxed in xylene and rehydrated in graded ethanol. Then, 3% hydrogen peroxide was used to inactivate endogenous enzymes. Tissue sections were immersed in citrate buffer and heated for antigen retrieval. After being blocked using 5% bull serum albumin, tissue sections were incubated with primary antibody, biotin–peroxidase secondary antibody, DAB reagents. Finally, tissue sections were counterstained with hematoxylin and photographed with a microscope.

### 2.10 Statistical analysis

Data management and analysis were performed with IBM SPSS 21.0 software. The data were normalized using mean± standard deviation for three independent experiments. Significance levels of measurement data were estimated using the student t-test or one-way ANOVA. *P* values <0.05 were deemed statistically significant.

## 3. Results

### 3.1 Reg3A expression between tumor and adjacent tissues

**Figure 1.**
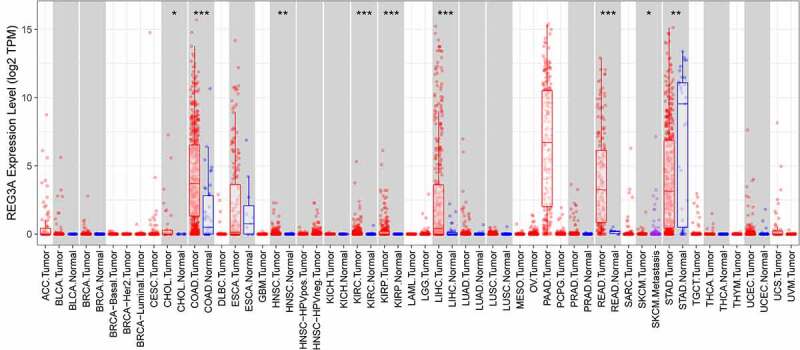
Reg3A expression in cancers in tumor immune estimation resource database. *, P < 0.05; **, P < 0.01; ***, P < 0.001

**Figure 2.**
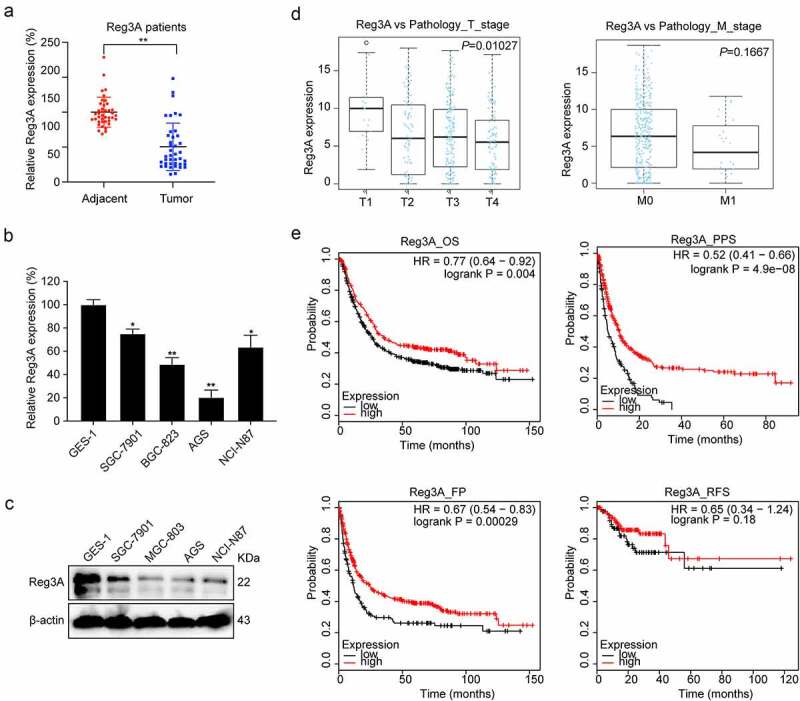
Reg3A expression between tumor and adjacent tissues. (a) Reg3A mRNA expression in GC patients. Reg3A (b) mRNA and (c) protein expression in GC cells. (d) The correlation between Reg3A and T stage, M stage in TCGA datasets. (e) Survival analysis of Reg3A in GC; *, P < 0.05; **, P < 0.01

In Tumor Immune Estimation Resource database, we found that Reg3A is decreased in GC tissues (Figure 1, *P* < 0.01). We verified the Reg3A mRNA expression levels in GC patients and cell lines. These results indicated that Reg3A is downregulated in GC patients (P < 0.01, Figure 2a) and cell lines (*P* < 0.05, Figure 2b). Besides, Reg3A protein expression is also decreased in GC cells (*P* < 0.05, Figure 2c).

### 3.2 Reg3A is correlated with clinicopathologic characteristics

There was a significant positive correlation between Reg3A expression and the T stage (*P* = 0.01027) but not with the M stage (Figure 2d) in TCGA datasets. Besides, we detected Reg3A mRNA expression levels using qRT-PCR in GC tissue including 40 samples and found that Reg3A mRNA expression levels are significantly associated with clinical characteristics, such as TNM classification, lymph node, and grade of differentiation. However, Reg3A mRNA expression levels are not significantly associated with tumor location, liver metastasis and peritoneal metastasis (Table 1). In the Kaplan Meier plotter database, survival analysis revealed that Reg3A significantly prolongs the OS (*P* < 0.01), PPS (*P* < 0.001), and FP (*P* < 0.001) of patients with GC. However, Reg3A did not prolong the RFS of patients with GC (Figure 2e).

**Table 1.** Association of Reg3A mRNA expression with clinicopathologic features in GC

Clinicopathologic Features	Cases	Reg3A mRNA (Mean±SD)	*P*
Age (years)			
<60	16	0.60 ± 0.37	0.1665
≥60	24	0.44 ± 0.31	
Gender			
Male	30	0.47 ± 0.29	0.205
Female	10	0.62 ± 0.46	
TNM classification			
I+ II	12	0.78 ± 0.35	<0.001
III+IV	28	0.39 ± 0.26	
LN metastasis			
Negative	12	0.78 ± 0.35	<0.001
Positive	28	0.39 ± 0.26	
Differentiation			
High differentiation	12	0.87 ± 0.35	<0.001
Moderately and poorly	28	0.35 ± 0.19	
Tumor location			
Fundus and cardia	7	0.49 ± 0.36	0.97
Body	11	0.52 ± 0.36	
Antrum	22	0.50 ± 0.34	
Liver metastasis			
Negative	34	0.53 ± 0.36	0.3288
Positive	6	0.38 ± 0.19	
Peritoneal metastasis			
Negative	31	0.54 ± 0.37	0.2634
Positive	9	0.39 ± 0.16	

lGC: gastric cancer; LN, lymph node.

### 3.3 Reg3A acts as a tumor suppressor in GC

**Figure 3.**
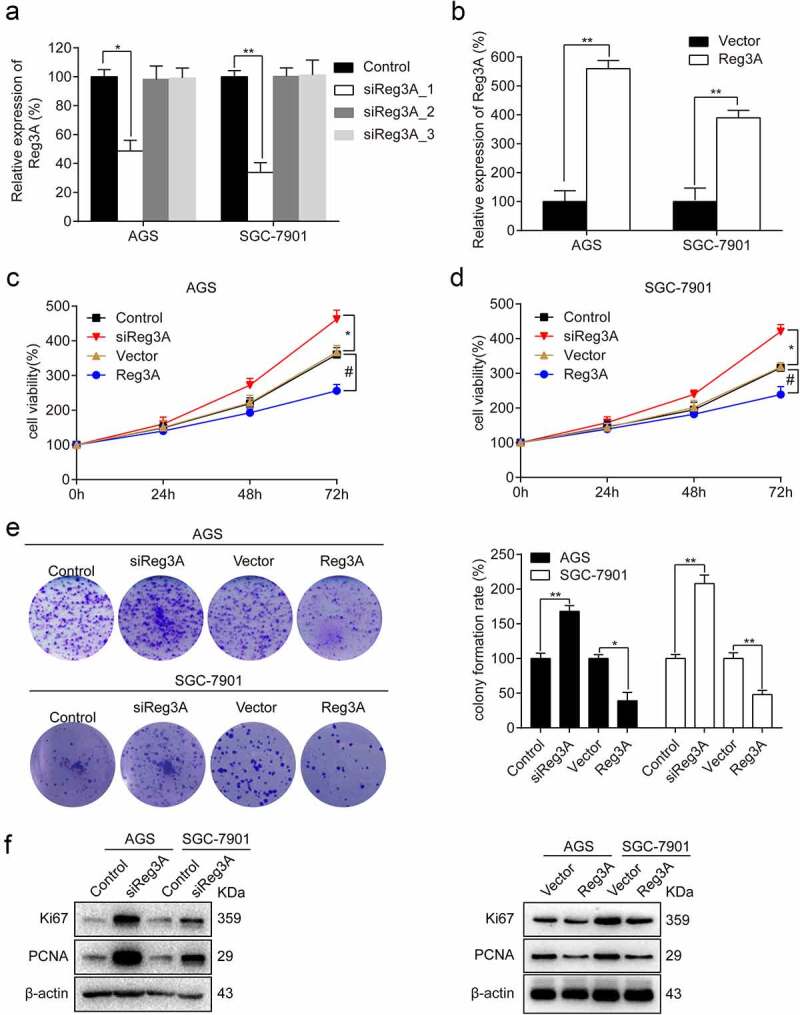
Reg3A inhibits cell proliferation in GC cells. Reg3A was (a) knocked down and (b) overexpressed. Reg3A regulates (c, d) cell viability, (e) clone formation, and (f) proliferation-related proteins. *, # P < 0.05; **, ## P < 0.01

To investigate the effect of Reg3A in GC, Reg3A was knocked down and overexpressed (*P* < 0.05, Figure 3a, b). Further analysis indicated that Reg3A siRNA significantly promotes cell viability, and overexpression of Reg3A significantly suppresses cell viability in AGS and SGC-7901 cells (*P* < 0.05, Figure 3c, d). Colony formation showed that Reg3A siRNA significantly increases colony formation rate (*P* < 0.01), and overexpression of Reg3A significantly decreases colony formation rate in AGS and SGC-7901 cells (*P* < 0.05, Figure 3e). Furthermore, Western blotting revealed that Reg3A siRNA significantly promotes Ki67 and PCNA expression, and Reg3A significantly inhibited Ki67 and PCNA expression (*P* < 0.05, Figure 3f).

### 3.4 Positive correlation between Reg3A and DMBT1

**Figure 4.**
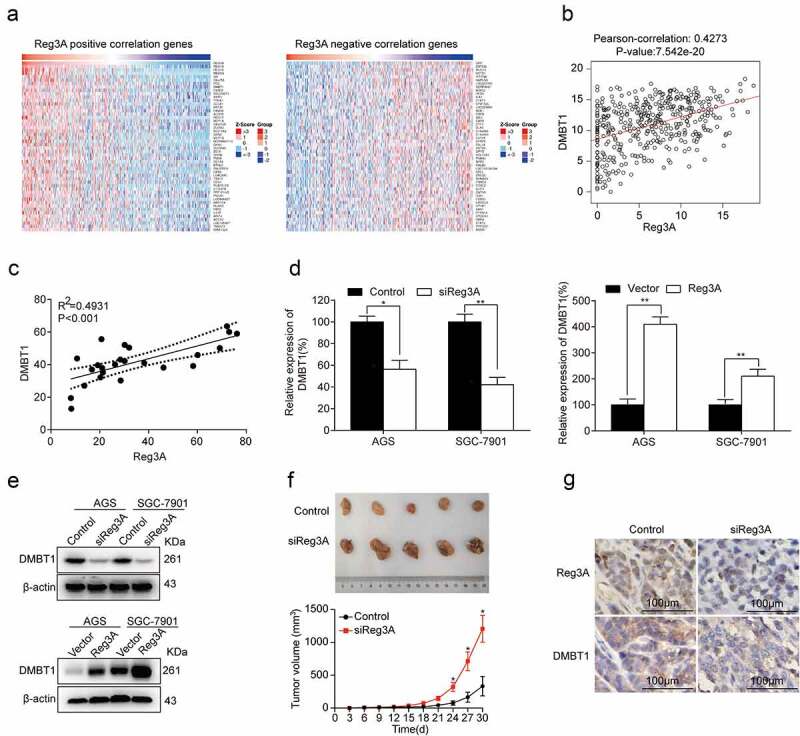
Reg3A regulated DMBT1 expression in GC. (a) Reg3A positive and negative correlated genes were required from LinkedOmics. (b) Reg3A was associated with DMBT1 in the TCGA database (P < 0.001). (c) The correlation between Reg3A and DMBT1 in GC patients. Reg3A regulates DMBT1 expression in (d) mRNA and (e) protein levels. (f) Xenograft tumorigenesis assay with AGS and AGS_siReg3A cells. (g) The expression levels of Reg3A and DMBT1 proteins in xenograft tumor tissues. *, P < 0.05; **, P < 0.01

The top fifty positively and fifty negatively correlated genes were required from LinkedOmics (*P* < 0.05, Figure 4a). Besides, Reg3A mRNA expression levels were significantly associated with DMBT1 mRNA expression levels in the TCGA database (*P* < 0.0001, Figure 4b) and patients (*P* < 0.001, Figure 4c). RT-qPCR assays reported that Reg3A positively regulates DMBT1 mRNA expression in GC cells (*P* < 0.05, Figure 4d). Western blot assays reported that Reg3A positively regulates DMBT1 protein expression in GC cells (*P* < 0.05, Figure 4e). Xenograft tumorigenesis assay found that siReg3A significantly promotes tumor growth (*P* < 0.05, Figure 4f). Immunohistochemistry assays demonstrated that siReg3A suppressed DMBT1 protein expression in tumor tissues (Figure 4g).

### 3.5 DMBT1 is downregulated in GC

**Figure 5.**
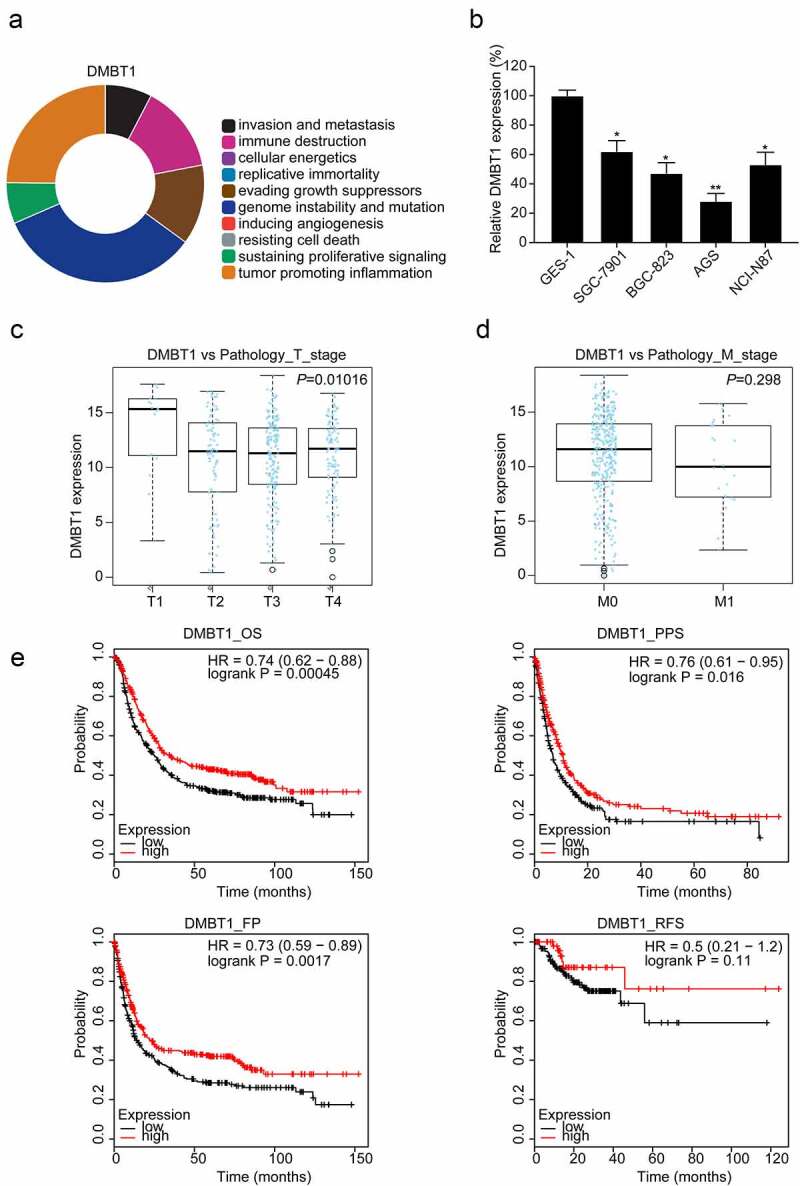
DMBT1 is downregulated in GC. (a) CHAT revealed the association between DMBT1 and hallmarks of cancer. (b) DMBT1 mRNA expression in GC cells. The correlation between DMBT1 and (c) T stage, (d) M stage in TCGA datasets. (e) Survival analysis of DMBT1 in GC. *, P < 0.05; **, P < 0.01

CHAT revealed that DMBT1 is mainly correlated with genome instability and mutation, tumor-promoting inflammation, immune destruction, and evading growth suppressors (Figure 5a). RT-PCR assays revealed that DMBT1 is also downregulated in GC cells (*P* < 0.05, Figure 5b). Besides, we found that DMBT1 mRNA expression levels are also significantly associated with the T stage (*P* < 0.05, Figure 5c), but not with the M stage (Figure 5d) in TCGA datasets. In the Kaplan Meier plotter database, survival analysis reported that DMBT1 also significantly prolongs the OS (*P* < 0.001), PPS (*P* < 0.05), and FP (*P* < 0.01, Figure 5e) of patients with GC. However, DMBT1 did not significantly prolong the RFS of patients with GC (Figure 5e).

### 3.6 Reg3A regulates cell proliferation by regulating DMBT1 expression

**Figure 6.**
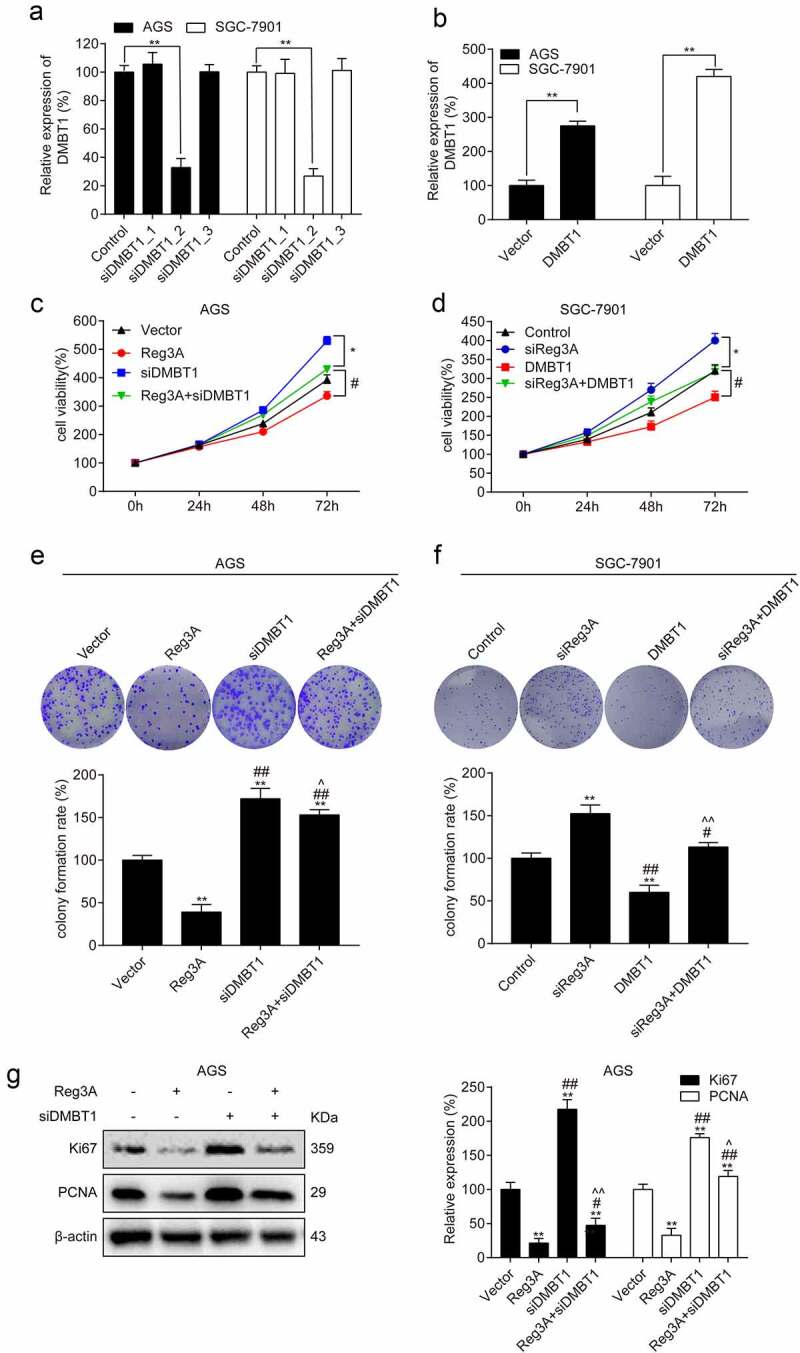
Reg3A regulated cell proliferation via DMBT1 in GC cells. DMBT1 was (a) knocked down and (b) overexpressed. Functions of Reg3A inhibiting cell viability (c) and clone formation (e) were reversed by DMBT1 siRNA. Functions of Reg3A siRNA promoting cell viability (d) and clone formation (f) were reversed by DMBT1. (g) Functions of Reg3A inhibiting Ki67 and PCNA expression were reversed by DMBT1 siRNA. *, P < 0.05 or **, P < 0.01 vs control or vector group; #, P < 0.05 or ##, P < 0.01 vs Reg3A or siReg3A group; ^, P < 0.05 or ^^, P < 0.01 vs siDMBT1 or DMBT1 group

DMBT1 was significantly knocked down and overexpressed in GC cells (*P* < 0.01, Figure 6a, b). The results showed that Reg3A significantly inhibited cell viability. Surprisingly, the effects of Reg3A inhibiting cell viability(*P* < 0.05, Figure 6c) and cell proliferation (*P* < 0.05, Figure 6e) were reversed by DMBT1 siRNA in AGS cells. Besides, the roles of Reg3A siRNA increasing cell viability (*P* < 0.05, Figure 6d) and colony formation rate (*P* < 0.05, Figure 6f) were reversed by DMBT1 in SGC-7901 cells. The functions of Reg3A inhibiting the expression of Ki67 and PCNA were reversed by DMBT1 siRNA in AGS cells (*P* < 0.05, Figure 6g).

## 4. Discussion

The effect of Reg3A on a variety of human cancers has been gradually recognized in recent years. Wang et al. found that Reg3A functions as a tumor promoter in pancreatic carcinoma, which is involved in the Reg3A-JAK2/STAT3 positive feedback loop [29]. However, the function and the downstream regulatory mechanism of Reg3A in GC remains elusive. Here, we confirmed that Reg3A mRNA and protein are downregulated in GC cells and tissues. We also analyzed the correlation between Reg3A and clinicopathologic features in GC (Table 1), which results indicated that Reg3A expression is negatively correlated with the grade of malignancy and the survival time of patients with GC. Hence, we hypothesized that Reg3A is a tumor suppressor in GC. Subsequently, we further analyzed the influence of Reg3A on cell growth in GC cells. Ki67 and PCNA are conventional proliferation-related markers [30]. Our findings suggested that Reg3A significantly suppressed Ki67 and PCNA expression in GC cells (Figure 3f), which indicated that Reg3A significantly inhibits cell proliferation. Next, we found that Reg3A positively regulates DMBT1 expression. DMBT1 also acts as a tumor suppressor in GC [23]. Hence, we hypothesized that Reg3A inhibits cell proliferation by targeting DMBT1 in GC. In Figure 6, we cotransfected Reg3A vector and DMBT1 siRNA, or Reg3A siRNA and DMBT1 vector in GC cells. The results indicated that DMBT1 acts as a target of Reg3A suppressing GC proliferation.

This study confirmed that Reg3A is a tumor suppressor in GC, which is consistent with that of Qiu et al. who revealed that Reg3A suppresses cell invasion, proliferation, and promotes apoptosis [6]. However, this finding is contrary to that of Chen et al. who found that Reg3A promotes cell proliferation, migration, and invasion [31]. The reasons for the conflict are still unclear; one possibility is the different cell lines in different. Chen et al. detected the basic expression levels of Reg3A in four GC cells. We noticed that Chen et al. did not set a control group, and MCF cells were derived from mice, which results cannot demonstrate their conclusion of ‘the expression of Reg3A is frequently increased in GC’. Besides, Chen et al. only knocked down Reg3A expression and did not overexpress Reg3A expression in GC cells. We consider that the results are insufficient to demonstrate the function of Reg3A in GC. We and Qiu et al. also analyzed the data in TCGA and GEO databases respectively, which results demonstrated our findings. Future large-scale clinical trials will help us verify Reg3A expression in GC.

Besides, we explored the potential correlate genes of Reg3A in the TCGA dataset (Figure 4a). We chose the top ten positively and ten negatively correlated genes and excluding the gene in Regenerating family members. We found that DMBT1 may be regulated by Reg3A after preliminary experiments. Our findings found that DMBT1 is downregulated in GC cells. However, Conde et al. reported that DMBT1 is frequently downregulated in well-differentiated gastric carcinoma but more frequently upregulated across various gastric cancer types [32]. Different stages of GC clinical samples may contribute to the discrepancy. Large-scale clinical trials and subgroup analysis will help us clarify the correlation between DMBT1 and the degree of tumor differentiation. Sousa et al. reported that DMBT1 is a biomarker for stomach metaplasia and gastric prognosis, and lower levels of DMBT1 correlated with more advanced diseases and a worse prognosis [33]. Bioinformatics analysis also found that DMBT1 reduces with the increase of the T stage in TCGA datasets (Figure 5c), and DMBT1 also significantly prolongs the OS, PPS, and FP (Figure 5e). These findings demonstrated that DMBT1 is a tumor suppressor in GC.

Remarkably, we found that Reg3A is relatively high expression in SGC-7901 cell, and is relatively low expression in AGS cell. So, we performed transfection experiments using AGS and SGC-7901 cells. Reg3A overexpression vector and DMBT1 siRNA were co-transfected into AGS cells. Reg3A siRNA and DMBT1 overexpression vectors were co-transfected into SGC-7901 cells. Our findings revealed that the function of Reg3A inhibiting cell growth is reversed by DMBT1 siRNA in AGS cells, and the function of Reg3A siRNA promoting cell growth was reversed by DMBT1 in SGC-7901 cells. These results revealed that Reg3A can target DMBT1 to regulate cell proliferation.

Based on the above results, this study first suggested that Reg3A can target DMBT1 to regulate cell proliferation in GC cells. Future studies will further determinate the regulatory mechanisms of Reg3A and DMBT1. A large-scale clinical trial will be conduct to verify the effect of Reg3A and DMBT1 on patients with GC.

## 5. Limitation of the study

GC contains various pathological classifications, such as adenocarcinoma, signet ring cell carcinoma, and undifferentiated carcinoma. Different types of GC have distinct incidence, clinicopathological features, treatments, and prognoses [34,35]. More experiments will be done to confirm or overturn these findings, which may help us distinguish between sensitive and nonsensitive groups for treatment.

## 6. Conclusion

DMBT1 acts as a novel target of Reg3A suppressing GC proliferation. Reg3A and DMBT1 may be potential biomarkers of diagnosis and prognosis in patients with GC.

## Highlights

Reg3A is down-regulated in GC.

Reg3A promotes DMBT1 expression.

Reg3A inhibits cell growth via DMBT1.
